# Pseudorabies virus uses clathrin mediated endocytosis to enter PK15 swine cell line

**DOI:** 10.3389/fmicb.2024.1332175

**Published:** 2024-02-05

**Authors:** Sabina Andreu, Carmen Agúndez, Inés Ripa, José Antonio López-Guerrero, Raquel Bello-Morales

**Affiliations:** ^1^Departamento de Biología Molecular, Universidad Autónoma de Madrid, Madrid, Spain; ^2^Centro de Biología Molecular Severo Ochoa (Consejo Superior de Investigaciones Científicas), Madrid, Spain

**Keywords:** pseudorabies virus, herpesvirus, clathrin, viral entry, PK15 cell line

## Abstract

Pseudorabies virus (PRV), a herpesvirus responsible for Aujeszky’s disease, causes high mortality in swine populations. To develop effective and novel antiviral strategies, it is essential to understand the mechanism of entry used by PRV to infect its host. Viruses have different ways of entering host cells. Among others, they can use endocytosis, a fundamental cellular process by which substances from the external environment are internalized into the cell. This process is classified into clathrin-mediated endocytosis (CME) and clathrin-independent endocytosis (CIE), depending on the role of clathrin. Although the involvement of cholesterol-rich lipid rafts in the entry of PRV has already been described, the importance of other endocytic pathways involving clathrin remains unexplored to date. Here, we characterize the role of CME in PRV entry into the PK15 swine cell line. By using CME inhibitory drugs, we report a decrease in PRV infection when the CME pathway is blocked. We also perform the shRNA knockdown of the μ-subunit of the adaptor protein AP-2 (AP2M1), which plays an important role in the maturation of clathrin-coated vesicles, and the infection is greatly reduced when this subunit is knocked down. Furthermore, transmission electron microscopy images report PRV virions inside clathrin-coated vesicles. Overall, this study suggests for the first time that CME is a mechanism used by PRV to enter PK15 cells and provides valuable insights into its possible routes of entry.

## 1 Introduction

Viruses may be simple in composition, but they have evolved to present a wide variety of complex mechanisms to interact with and infect host cells. Some viruses can enter the cell directly through fusion with the plasma membrane, but other routes based on endocytosis are preferred (Yamauchi and Helenius, 2013; Cossart and Helenius, 2014). Endocytosis is a fundamental mechanism described in eukaryotic cells, by which substances from the external environment (including solutes, fluids, components of the plasma membrane, and pathogens) are internalized into the cytoplasm through invagination of the cell membrane (Kaksonen and Roux, 2018). This process contributes to nutrient and ligand uptake, cell signaling, and adhesion, among others (Le Roy and Wrana, 2005).

Endocytosis can be classified into two main groups focusing on the role of clathrin, namely clathrin-mediated endocytosis (CME) or clathrin-independent endocytosis (CIE). The latter group includes caveolae-mediated endocytosis, lipid raft-dependent endocytosis, macropinocytosis and other lesser-known processes (Mercer et al., 2010; Hemalatha and Mayor, 2019). Although viruses primarily use CME as their route of entry, they can enter through more than one single pathway (Kaksonen and Roux, 2018; Ripa et al., 2021). Typically, the CME pathway begins with the stimulation by ligands of specific receptors on the cell membrane. This triggers the recruitment of triskelia-shaped clathrin to the plasma membrane forming clathrin-coated pits (CCPs) of 100 triskelia on average (Mettlen et al., 2018). Clathrin is anchored to the plasma membrane via protein adaptor complexes (such as adaptor protein 2 or AP-2), giving rise to clathrin-coated vesicles (CCVs) (McMahon and Boucrot, 2011; Brodsky, 2012; Mettlen et al., 2018). Finally, the GTPase activity of dynamin is responsible for the budding of clathrin vesicles by self-assembling into rings that form a collar around the neck of the vesicles (Mettlen et al., 2009; Prichard et al., 2022). Once the cargo has been internalized, not only the clathrin, but also the cytoplasmic complexes and membrane receptors involved in this process can be recycled and reused (Le Roy and Wrana, 2005; Barrow et al., 2013).

Pseudorabies virus (PRV) belongs to the family *Herpesviridae* and is the pathogenic agent of Aujeszky’s disease, which causes severe symptoms and high mortality in swine populations, its natural host (Andreu et al., 2020). Previous studies have demonstrated the entry of several herpesviruses through CME in different cell lines (Sobhy, 2017); for example, herpes simplex virus type 1 (HSV-1) (Nicola, 2016; Praena et al., 2020; Tebaldi et al., 2020), Epstein-Barr virus (EBV) (Millert and Hutt-Fletcher, 1992), bovine herpesvirus 1 (BoHV-1) (Pastenkos et al., 2018), and Kaposi’s sarcoma-associated virus (KSHV/HHV-8) (Kerur et al., 2010). In addition, the relevance of cholesterol-rich lipid rafts in PRV entry has already been characterized (Desplanques et al., 2008; Ren et al., 2011), but so far, the importance of other endocytic pathways that the virus hijacks to enter the cell has not been explored.

In the present study, we characterize the role of CME in the entry of PRV into the established swine cell line PK15. To this end, a set of studies with CME inhibitory drugs were performed, revealing a decrease in the infection when blocking this pathway by this method. The importance of clathrin in PRV infection was also determined by silencing the μ subunit of the adaptor AP-2, by a knockdown approach. Furthermore, transmission microscopy images also elucidate the interaction of PRV virions with clathrin vesicles. Our results provide the first evidence that the absence of CME reduces PRV infection and that this endocytic process is critical for PRV entry in PK15 cell line.

## 2 Materials and methods

### 2.1 Cell cultures

PK15 is an established cell line that originated from kidney epithelial cells of an adult pig (*Sus scrofa domestica*) (Todaro et al., 1974), and was generously provided by Dr. Yolanda Revilla (CBMSO, Madrid, Spain). Cells were maintained in low-glucose Dulbecco’s modified Eagle medium (DMEM) (Life Technologies, Paisley, UK) supplemented with 5% fetal bovine serum (FBS), glutamine (2 mM), penicillin (50 U/mL) and streptomycin (50 μg/mL) (Gibco, CA, USA), at 37 °C in a humidified atmosphere of 5% CO_2_. The absence of mycoplasma was confirmed by using the PCR Mycoplasma Detection kit (Takara Bio, San José, CA, USA).

### 2.2 Viruses

Recombinant strain PRV-XGF-N (Viejo-Borbolla et al., 2010) was kindly provided by Dr. Enrique Tabarés (UAM, Madrid, Spain). PRV-XGF-N was obtained by replacing the gene encoding the PRV gG glycoprotein (glycoprotein that is not part of the virion as it is secreted into the medium by infected cells, being not essential for virulence) with the *EGFP* gene in NIA-3 wild type PRV strain. PRV-XGF-N was propagated and titrated with a 50% tissue culture infective dose (TCID_50_) assay in PK15 cells as previously described (Andreu et al., 2021).

### 2.3 Antibodies and reagents

Chlorpromazine (C8138), dynasore (D7693), and pitstop 2 (SML1169) were purchased from Sigma-Aldrich (St. Louis, MO, USA). Mowiol was obtained from Calbiochem (Merck Chemicals, Darmstadt, Germany), To-Pro-3 from Thermo fisher (Waltham, MA, USA) and human transferrin (Tf) CF^®^543 and CF^®^555-Labeled Dye Dextran 10,000 MW conjugates were purchased from Biotium (Fremont, CA, USA). X-tremeGENE 360 Transfection Reagent (XTG360-RO) was obtained from Roche (Basel, Switzerland). All drugs in this study were dissolved in 0.1% v/v dimethyl sulfoxide (DMSO), except for chlorpromazine and Tf which were dissolved in sterile distilled water.

Primary antibodies used were mouse monoclonal anti-GFP (11814460001, Roche), mouse monoclonal anti-β-actin peroxidase antibody (A3854, Sigma), rabbit monoclonal anti-AP2M1 (ab75995, abcam, Cambridge, UK), and rabbit anti-IE180 (Gómez-Sebastián and Tabarés, 2004) (kindly provided by Dr. Enrique Tabarés). Horseradish peroxidase conjugate (HRP) secondary anti-IgG antibodies were purchased from Millipore (Darmstadt, Germany).

### 2.4 Cell viability assay

The potential cytotoxic effect of chlorpromazine, dynasore, and pitstop 2 in PK15 cells was analyzed by the MTT [3-(4,5-dimethylthiazol-2-yl)-2,5-diphenyltetrazolium bromide] assay (Promega, Cell Titer 96^®^ Non-Radioactive Cell Proliferation Assay). Non-confluent monolayers of PK15 cells plated in 96-well tissue culture dishes and cultured in DMEM supplemented with 5% FBS were incubated for 24 h with different concentrations between 0 and 100 μM of each compound. Four replicates were carried out for each concentration. Then, cells were incubated with a final concentration of 0.5 mg/mL of MTT in a humidified atmosphere for 4 h, and formazan crystals were solubilized in 0.01 M HCl with 10% SDS. The resulting-colored solution was quantified using the scanning multiwell spectrophotometer iMarkTM Microplate Reader (BioRad, Hercules, CA, USA), measuring the absorbance of formazan at 595 nm. The readouts obtained from MTT assay were further normalized to the value of untreated cells where the viability value was set to 100%.

The effect of the antibiotic puromycin on PK15 cells was also tested by this method. Cells were incubated for 48 h with puromycin ranging from 0 to 10 μM, and results were obtained following the same protocol as before.

### 2.5 Endocytosis assay

Cells grown on 24-well plates on round coverslips were treated for 1 h with either 20 μM chlorpromazine, 100 μM dynasore, or 50 μM pitstop 2 at 37°C and then maintained for 30 min on ice with human transferrin conjugate Tf CF^®^543 (5 μg/mL) or CF^®^555 Labeled Dye Dextran 10,000 MW (5 μg/mL). All cells were incubated again for 5 or 10 min, respectively, at 37°C in a humidified 5% CO_2_ atmosphere to allow internalization of Tf or dextran conjugate. Finally, cells were washed with PBS followed by fixation for immunofluorescence microscopy.

### 2.6 Immunofluorescence microscopy

Cells grown on 24-well plates on round coverslips were fixed in 4% paraformaldehyde (PFA) for 15 min and rinsed with PBS. Cells were then permeabilized with 0.2% Triton X-100, rinsed and incubated for 30 min at room temperature (RT) with 3% bovine serum albumin in PBS (blocking buffer). For a labeled immunofluorescence analysis, coverslips were incubated in a wet chamber and nuclei were stained with To-Pro-3 for 10 min. After thorough washing, coverslips were mounted on a slide using mounting media (Mowiol). Images were obtained using a LSM 710 Inverted Confocal Microscope (Zeiss, Vienna, Austria), equipped with an Argon laser and a He/Ne 633 nm laser, using 40x/1.4 NA Oil lens. Pinhole size was set to 1 AU. Processing of confocal images was performed using the Fiji-ImageJ software (version Image J 1.53c) (Schindelin et al., 2012).

### 2.7 Flow cytometry

To perform fluorescence activated cell sorting (FACS) analysis, cells were dissociated by 2 min incubation with 0.05% trypsin/0.1% EDTA (Invitrogen, Waltham, MA, USA) at RT and washed and incubated for 20 min with Ghost Dye Red 780 (1:1000 in PBS, CYTEK). After centrifugation, the pellet was washed with PBS and fixed with 1%-PFA-1% FBS in PBS for 15 min. Finally, cells were rinsed and resuspended in PBS. Cells were analyzed using a FACSCalibur Flow Cytometer from BD (Franklin Lakes, NJ, USA). Data were processed with FlowJo software (BD, version 10.6.2).

### 2.8 Western blot analysis

Cells were lysed using the radioimmunoprecipitation assay (RIPA) buffer mixed with a protease inhibitor cocktail from Roche (Basel, Switzerland), and the total protein load was quantified by Bradford assay (Bio-Rad Laboratories, Inc. Hercules, CA, USA). Equalized protein samples were separated by SDS-PAGE in 10% acrylamide gels under non-reducing conditions and later transferred onto Merck Millipore Immobilon-P membranes. Membranes were blocked for 30 min in 5% non-fat dry milk at RT, incubated overnight at 4°C with the appropriate primary antibodies (anti-GFP 1:1000, anti-IE180 1:200), washed with 0.05% Tween 20 in PBS, and incubated with HRP-conjugated secondary antibodies for 1 h at RT. Anti-β-actin-peroxidase antibody (1:50,000) was directly incubated for 1 h at RT. After extensive washing, the membranes were finally incubated with an enhanced chemiluminescence Western blotting kit, ECL Western Blotting Detection Reagent (GE. Healthcare, Chicago, IL, USA) to visualize the protein band. The intensity of immunoblot bands was analyzed using Fiji-ImageJ software (version Image J 1.53c).

### 2.9 shRNA knockdown

To knockdown (KD) the gene expression of AP2M1 in PK15 cells, the RNA interference strategy was used. Five commercial shRNA oligonucleotides designed to target against different regions of the AP2M1 gene were purchased (Table 1). Control cells were transfected with pLKO.1-puro non-target shRNA control plasmid DNA (Sigma-Aldrich).

**TABLE 1:** ID and specific sequence of commercial shRNA plasmid DNAs (Sigma-Aldrich) used for the KD of AP2M1 gene in PK15 cell line.

ID of shRNA plasmid DNA	Code name	Specific sequence
TRCN0000060238	A	5′-GTGGTCATCAAGTCCAACTTT-3′
TRCN0000060239	B	5′-CACCAGCTTCTTCCACGTTAA-3′
TRCN0000060241	C	5′-GCTGGATGAGATTCTAGACTT-3′
TRCN0000333063	D	5′-GTGGTCATCAAGTCCAACTTT-3′
TRCN0000381904	E	5′-GGCGAGAGGGTATCAAGTATC-3′
SHC016-1A	Non-target	5′-GCGCGATAGCGCTAATAATTT-3′

PK15 cells were seeded in 96-well tissue culture plates at an approximate confluency of 70%. At least 1 h after cell transfection, the culture medium was replaced for fresh DMEM medium supplemented with 5% FBS. shRNA plasmid DNAs were diluted in DMEM to reach a concentration of 1 μg/μl. A total of 100 μl of this shRNA was mixed with the XtremeGENE transfection agent (3 μl of reagent for every 100 μg of shRNA; ratio 3:1) and this mixture was incubated for 20 min at RT. Subsequently, 10 μl of said mix was added in the form of drops to 10 wells per condition and the plate was shaken vigorously to ensure distribution over the entire surface. After 48 h of incubation at 37°C in a humidified atmosphere of 5% CO_2_, the transfection mix was replaced with DMEM supplemented with 5% FBS and 5 μg/ml puromycin to select transfected cells. Two days later, cells growing in medium with puromycin were isolated, grown on 24-well plates, and processed for immunoblot or RT-qPCR to determine the efficacy of shRNA silencing. A previous cytotoxicity assay (MTT) was performed to elucidate the concentration of puromycin that was able to kill wild type PK15 cells which had not incorporated the plasmid, as described in the “cell viability assay” section.

### 2.10 RT-qPCR

To characterize the success in silencing *AP2M1* gene in PK15 cells, transfected cells A6, B3 and C1 were cultured in 24-well plates for 24 h. Later, cells were treated with 0.25 trypsin and 0.03% EDTA in PBS and the total RNA from six different samples per condition was extracted using a RNeasy mini kit (Qiagen, Venlo, Netherlands). RNA integrity was evaluated on an Agilent 2100 bioanalyzer (Agilent Technologies, Santa Clara, CA, USA), and quantification of RNA was performed on a Nanodrop ND-1000 spectrophotometer (Thermo Fisher). RNA integrity number (RIN) values were between 9 and 10, corresponding to samples with high integrity. Genomic DNA contamination was assessed by amplification of representative samples without retrotranscriptase. RT reactions were performed using SsoFast™ EvaGreen^®^ Supermix (Bio-Rad, #1725204) and qPCR using Power SYBR^®^ Green PCR Master Mix (ThermoFisher, #4367659). Briefly, 1 μg of total RNA from each sample was combined with master mix containing a mixture of random primers and oligo-dT for priming. The No-RT master mix included in the pack was used as RTcontrol. The reaction volume was completed up to 20 μL (RT reactions) and 10 μL (qPCR) with DNAse/RNAse free distilled water following manufacturer’s instructions. Amplification conditions of qPCR followed these steps: (30 s × 95°C, 5 s × 60°C) × 40 cycles and melting curve from 60 to 95°C (incrementing 0.5°C/s). Amplifications of the representative samples were either negative or delayed more than 5 cycles compared to the corresponding RT + reactions. Primers were designed by the Genomics Core Facility at the Centro de Biología Molecular Severo Ochoa (CBMSO) using Primer Blast, following an intron-spanning strategy. To guarantee silencing efficacy, *AP2M1* primer sets were designed to overlap the site targeted by the shRNAs on the mRNA. The NormFinder algorithm was used to identify β-actin (*ATCB*) as the most suitable gene for the normalization due to its high stability. Primer sequences were as follows (Table 2).

**TABLE 2:** ID and specific sequence of customized primers used for qPCR.

Primer set	Forward	Reverse
A6	5′-TCCGAGTGATCCCGCTAGT-3′	5′-TTCTCGCTGGCCTTGTACTT-3′
B3	5′-CGGGTCTACCGAGATGACA-3′	5′-TCGAAGACCATGGCAGCATT -3′
C1	5′-GTGTGATGTAATGGCTGCCT-3′	5′-CTTCTTTCGTCTGATGCTGGCT-3′
WT	5′-GGCATCAAAAGCCAGCATC-3′	5′-GGAGGTTCACACTCTCCAGC-3′
ACTB	5′-CCTCCTTCCTGGGCATGG-3′	5′-GGATGCTCCATCCAACCGAC-3′

### 2.11 Transmission electron microscopy (TEM)

Cells were seeded in 24-well tissue culture plates and maintained in DMEM supplemented in 5% FBS for 24 h. Then, they were pretreated or not with 100 μM dynasore, 20 μM chlorpromazine or 50 μM pitstop 2 for 1 h before infection, and then infected or mock-infected with PRV-XGF-N at an m.o.i of 30 for 1 h at 4°C, to allow viral attachment to the cellular surface without yet entering the cell. After this time, cells were incubated at 37°C for 20 min. Finally, cells were fixed with 2% PFA+ 2.5% glutaraldehyde in 0.1 M phosphate buffer, pH 7.4 and processed for transmission electron microscopy. Fixed monolayers were washed once with PBS and once with distilled water. Cells post-fixation was as follows: 45 min at RT with 1% osmium tetroxide (TAAB Laboratories Equipment Ltd.) in PBS, washed with distilled water, 45 min incubation ay RT with 1% aqueous uranyl acetate (Electron Microscopy Sciences, Hatfield, PA, USA) and after dehydration with increasing concentrations of ethanol absolute, cells were embedded in epoxy resin EML-812 (TAAB Laboratories Equipment Ltd., 2 days, RT). Resin-containing gelatin capsules (TAAB Laboratories Equipment Ltd.) were placed on the coverslips and polymerized (2 days, 60°C). Resin blocks were detached from coverslips by successive immersion in liquid nitrogen and hot water. Ultrathin 70 nm-thick sections, parallel to the monolayer, were obtained with a Leica ultramicrotome (Leica Microsystems GmbH, Wetzlar, Germany), transferred to Formvar-coated EM GS2x1-N3 nickel buttonhole grids, and stained with 5 % aqueous uranyl acetate (10 min, RT) and lead citrate (3 min, RT). Sections were visualized on a JEOL 1400 electron microscope equipped with a LaB6 filament and operated at 100 kV. Images were recorded with a GATAN One View digital camera at various magnifications and processed by Fiji-ImageJ software (version Image J 1.53c).

### 2.12 Statistical analysis and quantification of fluorescence

Results obtained from the experiments were analyzed using Prism software v8.0.1 (GraphPad software, Inc., San Diego, CA, USA). Data were subjected to Mann-Whitney *U*-tests to determine significant differences between groups, and a *P*-value of < 0.05 was considered statistically significant. For fluorescence intensity quantifications, various regions of interest (ROIs) were measured from groups of 30 cells with 3 areas of each image to normalize for the cell number and background intensity. The software Fiji-ImageJ (version Image J 1.53c) was used to quantify fluorescence intensity for the 555 channel. Images were transformed to 8-bit gray scale and fluorescence intensity was analyzed using the particle analysis function, after correcting the background, as previously described (Shihan et al., 2021).

## 3 Results

### 3.1 Endocytosis assays with CME chemical inhibitors and its cytotoxic effect in PK15 cell line

**FIGURE 1:**
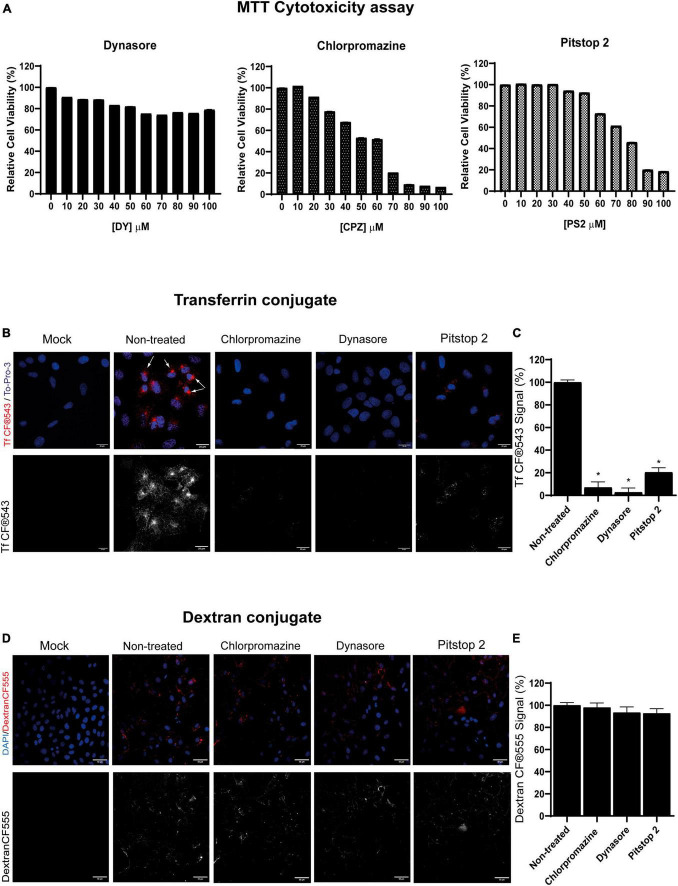
Clathrin-mediated endocytosis (CME) chemical inhibitors dynasore, chlorpromazine, and pitstop 2 disrupt transferrin but no dextran uptake at non-cytotoxic doses. **(A)** Cellular viability of PK15 cells exposed to dynasore, chlorpromazine, and pitstop 2 for 24 h. Cell viability was measured by MTT tetrazolium salt assay and calculated as the percentage of cell viability compared to untreated cells; columns represent the mean percentage of relative cellular viability ± S.D. (*n* = 4) after exposure to the drugs. **(B)** Transferrin uptake in PK15 cells is blocked by CME chemical inhibitors. PK15 cells were mock-treated or treated with either 20 μM chlorpromazine, 100 μM dynasore, or 50 μM pitstop 2 for 1 h and then incubated for 30 min on ice with Tf CF^®^543 (5 μg/ml). Finally, cells were fixed after 5 min of incubation at 37°C. Fluorescence microscopy images are shown, with Tf in red and cellular nuclei stained with To-Pro-3 in blue. Arrows point to the accumulation of Tf in the endosomal recycling compartments. **(C)** Quantification of Tf CF^®^543. Cells were acquired and analyzed as in **(B,D)**; ROIs from groups of 30 cells and 3 areas of each image were measured. Mean percentage of fluorescence ± S.D. is shown. **(D)** Dextran-CF^®^555 uptake is not altered in PK15 cells treated with CME-inhibitory drugs. PK15 cells were incubated or mock-incubated as described in **(B)** but instead of Tf, they were incubated with CF^®^555 Labeled Dye Dextran 10,000 MW (5 μg/mL) for 15 min at 4°C. Cells were then transferred to 37°C for 10 min, washed, and then fixed with 4% PFA and stained as before. **(E)** Quantification of CF^®^555 Dextran. Cells were acquired and analyzed as previously described. Measurement of mean fluorescence intensity from 555 channel in a ROI was performed. Mean percentage of fluorescence ± S.D. is shown.

First, CME chemical inhibitors chlorpromazine, dynasore and pitstop 2 were tested to determine the concentrations that did not exhibit harmful cytotoxic effects in PK15 cells. After 24 h of drug addition, cells maintained their viability above 70% when treated with either 100 μM dynasore, 20 μM chlorpromazine, or 50 μM pitstop 2 (Figure 1A). This was the threshold chosen for the rest of the assays, as recommended by the ISO 10993-5:2009 (E) standard (ISO 10993-5:2009, Under Review). The absence of cytotoxicity of the solvent at the concentrations used to make the pertinent dilutions of the drugs (DMSO) was also demonstrated (results not shown).

It is well-known that human transferrin uses the CME pathway to enter the cells (Cossart and Helenius, 2014). In this term, human transferrin conjugate Tf CF^®^543 was used in this study to establish a suitable method to monitor CME endocytosis in PK15 cells and to demonstrate that the compounds dynasore, chlorpromazine and pitstop 2 did block this pathway. These three drugs are known for blocking CME by using different strategies: dynasore blocks the GTPase dynamin that leads to the fission of CCPs from the plasma membrane (Macia et al., 2006); pitstop 2 prevents clathrin-heavy-chain (CHC) from interacting with adaptor proteins necessary for the formation of CCPs (Bayati et al., 2021), and chlorpromazine dissociates clathrin networks in the inner leaflet of the plasma membrane during CCPs assembly (Vercauteren et al., 2010), all blocking the uptake of Tf.

Cells were pretreated for 1 h with dynasore, chlorpromazine or pitstop 2 at 37°C and then incubated for 30 min on ice with Tf conjugate (5 μg/mL). Subsequently, cells were incubated again for 5 min at 37°C in a humidified 5% CO_2_ atmosphere to allow internalization of Tf (still in the presence of the inhibitors), and finally cells were processed for confocal microscopy. Fluorescence microscopy images after treatment with the drugs showed a decrease in Tf uptake, in comparison to the non-treated control (Figure 1B). Quantification of Tf uptake is also performed (Figure 1C). These drugs reduced the internalization of Tf, suggesting that CME was partially blocked or inhibited. Furthermore, no appreciable amounts of Tf were observed in dynasore-treated cells, but a small amount of Tf is noticeable in the case of chlorpromazine and even more in pitstop 2-treated cells. However, the simulated CME is insignificant compared to non-treated cells, in which there is a marked accumulation of Tf in a perinuclear region, corresponding to the endosomal recycling compartment (Figure 1B, marked with arrows). In addition, CME drugs showed a dose-dependent effect on the inhibition of Tf uptake, with the highest non-cytotoxic concentrations inhibiting the most (results not shown).

Furthermore, to confirm that the drugs used in this study did not interfere with other endocytic pathways at the concentrations tested, the effect on the uptake of fluorescently labeled dextran, which is internalized following a clathrin-independent route, was analyzed (Figure 1D). All drugs blocked internalization of Tf, a process that requires formation of CCPs and is dynamin dependent (Cossart and Helenius, 2014), while maintaining internalization of dextran, whose internalization was also quantified (Figure E).

### 3.2 Effect of CME inhibitory drugs in PRV infection

**FIGURE 2:**
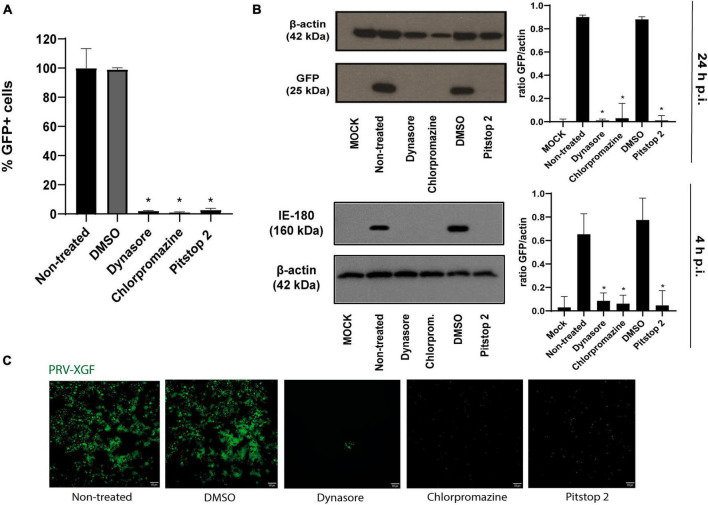
Pseudorabies virus (PRV)-XGF-N infection is blocked by CME chemical inhibitors. PK15 cells were pre-treated with either 0.1% (v/v) DMSO, 100 μM dynasore, 20 μM chlorpromazine, or 50 μM pitstop 2 for 1 h and then infected with PRV-XGF-N at an m.o.i of 0.5 (collected at 24 h p.i.) or moi 5 (collected at 4 h p.i.). Cells were maintained until they were collected in the presence of the drugs. **(A)** Flow cytometry data show the percentage of normalized infection 24 h p.i. (% GFP+ cells) ± S.D. for each condition. Triplicate experiments were performed for each data point (*n* = 3). *p < 0.05. **(B)** Western blot analysis of total cell lysates showing viral GFP and IE180 for each condition. β-actin was used as protein loading control. Values of Western blot quantification are reported as the mean ± S.D. (*n* = 3), *p < 0.05. **(C)** Fluorescence microscopy images show GFP+ signal corresponding to viral infection 24 h p.i. (*n* = 3).

Once the efficacy of the drugs in blocking CME in PK15 cell line was determined, the next step was to study whether this inhibition of the route affected PRV infection. For this, the internalization of PRV-XGF-N was examined in the presence of dynasore, chlorpromazine, and pitstop 2. Briefly, PK15 cells were pre-treated for 1 h with these drugs and then infected with PRV-XGF-N at an m.o.i of 0.5 for another hour. Cells were maintained in culture medium after infection and samples were collected at 4 or 24 h p.i for flow cytometry, Western blot, and confocal fluorescence microscopy. A significant decrease in viral infection was noticed in all cells pretreated with CME-blocking drugs, in comparison to non-treated and DMSO-treated cells. This decrease in viral GFP was detected not only in flow cytometry analysis (Figure 2A), but also in Western blot (Figure 2B), where no band corresponding to viral GFP is revealed in the mentioned conditions. For samples collected at 4 h p.i., the expression of immediate protein IE180 was evaluated (Figure 2B), to ensure that the decrease in infection was due solely to clathrin blockade during the entry phase. It is when PRV has already entered, and the genome arrives to the nucleus when IE genes are transcribed. As in the case of GFP, no band corresponding to viral IE180 is observed in the drug-treated samples. The same decrease in viral GFP is reported in fluorescence microscopy images (Figure 2C). These results suggest that CME could be a possible route of entry of PRV.

### 3.3 Effect of AP2M1 knockdown silencing in PRV infection

**FIGURE 3:**
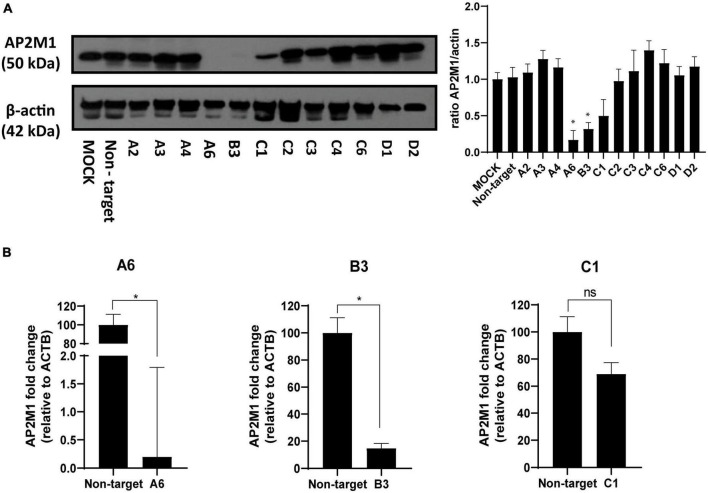
Determination of AP2M1 shRNA knockdown efficiency in PK15 cells. PK15 cells were transfected with a pool of shRNA to KD the expression of AP2M1. KD efficiency was checked by both Western blot and RT-qPCR. **(A)** Western blot analysis of total cell lysates subjected to SDS-PAGE showing AP2M1 for each condition. β-actin was chosen as protein loading control. Values of immunoblot quantification are reported as the mean ± S.D. (*n* = 3); *p < 0.05. **(B)** qPCR amplification of AP2M1 mRNA from AP2M1 A6, B3, and C1 shRNA-treated PK15 cells in comparison to the non-targeted control. mRNA level is relative to β-actin (*ACTB*) control (*n* = 6, mean ± S.D.). *p < 0.05 was considered as statistically significant compared to the non-targeting shRNA-transfected cells.

A loss-of-function analysis was performed to inhibit CME by knocking down the gene encoding the subunit μ of the AP-2 (AP2M1) adaptor with a previously defined shRNA pool. This could best demonstrate the role of clathrin in PRV entry, as the AP-2 complex plays an essential role in CME (Rappoport, 2008). PK15 cells were transfected with various shRNA plasmid DNAs targeting different sites located on the CDS of AP2M1 and were selected at 48 h post-transfection in a media supplemented with 5 μg/ml puromycin. From 5 different shRNA initially used, only three shRNAs A, B, and C (see Table 2, “2 Materials and methods”) kept viable, and the efficacy of shRNA silencing was evaluated in these samples. Multiple shRNA for the same target were used to verify that the correct phenotype caused by the KD is observed. Transfection of PK15 cells with AP2M1 shRNAs led to a decrease in the expression of AP2M1 protein levels in three of the samples (A6, B3, C1) when compared to cells transfected with non-target shRNA, as demonstrated by Western blot analysis (Figure 3A). RT-qPCR analysis was also made to amplify the target mRNA (Figure 3B), using flanking primer sets that encompassed the region containing the shRNA target. Successful KD of AP2M1 in samples A6 and B3 was achieved, as seen by the reduced levels of mRNA, so this pair of transfected cells were the ones selected for further studies. However, the KD of AP2M1 in C1 PK15 cells was not significantly achieved; the decrease in AP2M1 mRNA relative levels was approximately 30%, and a band of little intensity referring to AP2M1 is shown in Western blot analysis (protein expression levels). Furthermore, no differences either in shape or size (Supplementary Figure 1A) or in viability (Supplementary Figure 1B), were observed between wild-type PK15 and PK15 AP2M1-KD cells (which supports that cells might also use CIE routes to maintain a normal surface/volume ratio). To further confirm that the CME pathway was effectively blocked in KD cells, A6, B3, and C1 were incubated with Tf conjugate, revealing a decrease in the internalization of this compound in successfully KD-samples compared to wild-type PK15 cells (Supplementary Figure 2).

**FIGURE 4:**
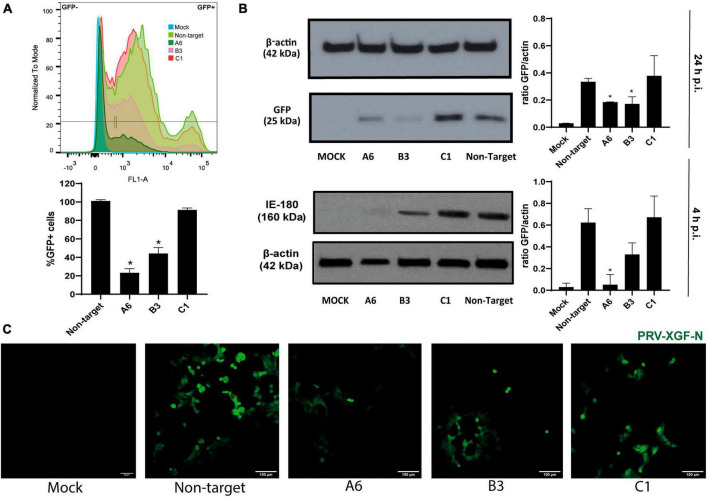
The knockdown of *AP2M1* in PK15 cells reduces PRV infection. KD PK15 cells A6, B3, and C1, and non-target cells were subjected to infection with PRV-XGF-N at an m.o.i of 0.5 (collected at 24 h p.i.) or moi 5 (collected at 4 h p.i.). After the corresponding time, samples were processed for the following experiments. **(A)** Flow cytometry analysis show the mean of the percentage of normalized infection 24 h p.i. (%GFP+ cells) ± S.D. (*n* = 4). Plots represent the histograms of GFP-positive (+) and GFP-negative (-) cells; *p < 0.05. **(B)** Western blot analysis of total cell lysates subjected to SDS-PAGE showing viral GFP or IE180 for each condition. β-actin was chosen as protein loading control. Values of immunoblot quantification are reported as the mean ± S.D. (*n* = 3); *p < 0.05. **(C)** Fluorescence microscopy images show GFP+ signal corresponding to viral infection 24 h p.i.

After verifying the specific KD of AP2M1 in PK15 cells, successfully KD samples were infected with PRV-XGF-N to study whether the lack of the μ subunit of AP-2 affects the entry of this virus. As it was shown above, A6 was the sample showing the lowest expression of AP-2, followed by B3 and finally C1. Flow cytometry analysis (Figure 4A) reported that GFP corresponding to viral infection decreased the most in A6 cells, followed by B3 and C1, the latter reaching the same infection levels as non-target cells. These results were corroborated by immunoblot (Figure 4B), and fluorescence microscopy (Figure 4C). The expression of IE180 at short times after infection was also performed (Figure 4B), following the same pattern of expression as GFP at 24 h p.i.. Furthermore, the decrease in infection rates tended to follow a directly proportional relation with the expression of AP2M1; the lower the AP2M1 expression, the lower the viral infection rate. This experiment was also performed with PRV wild-type strain NIA-3, following the same methodology as with PRV-XGF-N (Supplementary Figure 3), to ensure that the recombinant strain used during all experiments behaved the same way than the wild-type one. According to the results, AP2M1 knockdown reduces PRV-XGF-N endocytosis into PK15 cells.

### 3.4 Visualization of clathrin-coated vesicles and their interaction with PRV by transmission electron microscopy

**FIGURE 5:**
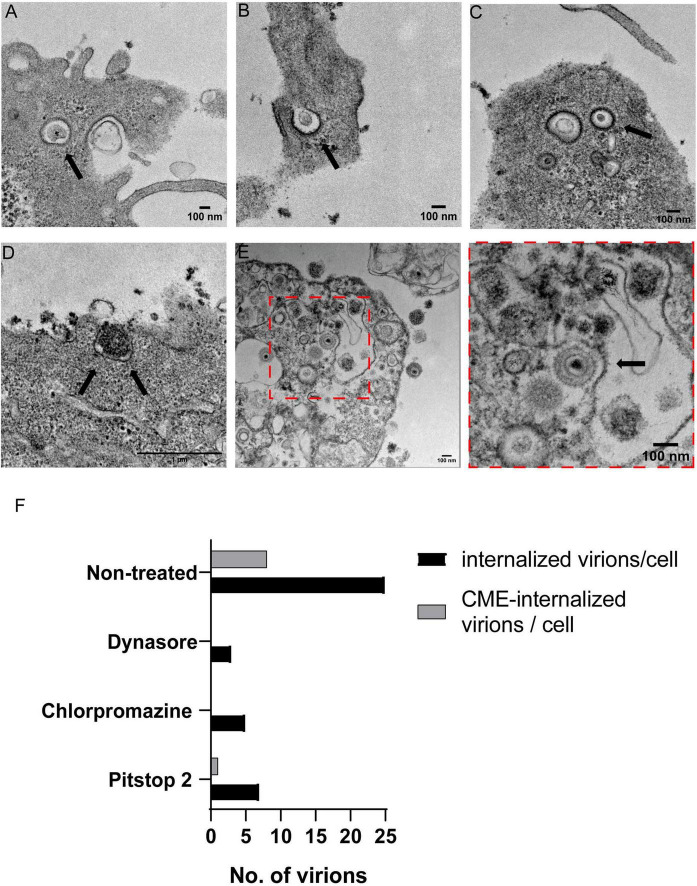
Transmission electron microscopy analysis of PRV-XGF-N virions inside clathrin vesicles in PK15 cells. Cells were infected with PRV-XGF-N at an m.o.i of 30 for 1 h at 4°C. Then they were maintained for 20 min at 37°C and processed for TEM. Arrows point to in **(A–C)** PRV-XGF-N virions inside clathrin vesicles, **(D)** virion entering a forming clathrin vesicle anchored in the cell membrane, and **(E)** overview of PRV-XGF-N surrounded by clathrin. **(F)** Quantification of the number of internalized virions per cell (*n* = 20 cells analyzed) in cells non-treated or treated with 100 μM dynasore, 20 μM chlorpromazine or 50 μM pitstop 2 for 1 h before infection, and during the 20 min of infection. The graph shows the number of virions inside the cell and from those, the virions entering via CME or internalized into CCVs.

Transmission electron microscopy (TEM) assays were performed to ultra-structurally analyze how PRV entered PK15 cells in a CME-dependent pathway. Fortunately, clathrin is naturally electrodense and can be directly seen without immunolabeling. TEM images revealed PRV-XGF-N virions internalized in CCVs (Figures 5A–C), directly being surrounded by clathrin anchored to the cell membrane (Figure 5D) or surrounded by clathrin (Figure 5E). Clathrin vesicles are characterized for showing its characteristic “bristle-like” pattern. Quantification of the number of virions internalized through CME was also assessed (Figure 5F), revealing that in all samples treated with CME-blocking drugs, the number of internalized virions was lower, and from those that managed to enter the cell, no viruses were detected using CCVs to enter. These results in concordance with the above strongly suggest that CME is clearly involved in the entry of PRV in PK15 cell line.

## 4 Discussion

Based on the available evidence, PRV can enter different cell types by using distinct mechanisms. Previous studies have reported non-endocytic pathways for PRV entry in RK13 cells (Granzow et al., 2005) and CHO cell lines (Nixdorf et al., 1999), and fusion under low pH conditions in PK15, Vero, MDBK and SK-N-SH cell lines (Miller et al., 2019). In addition, hypertonic treatment in PK15 infected cells (to inhibit viral entry via endocytosis in a general way) revealed a decrease in PRV infectivity, suggesting that this virus might use endocytic-related pathways to enter this cell line (Miller et al., 2019). Another recent study in which researchers inhibit CME with an inhibitor of Niemann-Pick C2 (NPC1) results in defects in CCP dynamics, which acutely interfere with Tf endocytosis, and PRV entry (Li et al., 2022). Nevertheless, no further research on whether PRV uses CME to enter PK15 cells has been done to date.

Depletion of essential proteins that mediate CME derive into indirect effects on cellular function and/or morphogenesis (Dutta et al., 2012). For this reason, selective inhibitory drugs have been used in this study, as a first pharmacological approach, revealing a decrease in the infection with PRV-XGF-N when blocking CME. On the one hand, dynasore is a reversible inhibitor of the GTPase activity of dynamin, preventing the fission of CCPs (Kirchhausen et al., 2008), and pitstop 2 prevents the interaction between CHC and adaptor proteins related with formation of CCPs (Bayati et al., 2021). Nonetheless, the pleiotropic and off-target effects of these drugs have been described, as dynasore also presents dynamin-independent effects such us the disruption of lipid rafts, and pitstop 2 was reported to have additional cellular targets besides CHC (Macia et al., 2006; Dutta et al., 2012; Preta et al., 2015). On the other hand, chlorpromazine specifically inhibits CME by dissociating clathrin networks in the inner leaflet of the plasma membrane during CCPs formation (Vercauteren et al., 2010). To validate the endocytosis assay performed with these drugs, we monitored the entry of dextran sulfate, a compound that is internalized by a clathrin-independent route. As a result, at the concentrations and cell system used here, while all CME inhibitory drugs altered Tf uptake, there were no differences in dextran uptake.

To confirm the results obtained in the experiments performed in the presence of the drugs, silencing of the μ subunit of AP-2 (AP2M1) with shRNAs was additionally performed. AP-2 is the second most abundant component of clathrin vesicles. This adaptor plays an important role in the maturation of CCPs so, when it is not present, the clathrin is not recruited and mature vesicles do not form (McMahon and Boucrot, 2011). AP-2 is an heterotetramer formed by four subunits (α, β, σ, and μ) and the phosphorylation of μ subunit by the kinase enzyme AP-2 associated protein kinase 1 (AAK1) stimulates clathrin and supports the cell surface receptor incorporation (Robinson, 2015; Tongmuang et al., 2020). As no chemical inhibitors of the whole AP-2 adaptor are commercially available, we used shRNA-based KD of the subunit μ, achieving approximately 90% depletion. AP-2 is structured into two heterodimers α/σ and ß/μ, so the lack of μ inhibits the assembly of heterodimer ß/μ and then leads to degradation of the ß subunit. As not enough ß/μ heterodimer is available, the full assembly is stopped and degradation of the other dimer α/σ is induced (Tobys et al., 2021). Several studies report that AP-2 complex acts, through its μ subunit, as a physical linker between clathrin triskelions and internalization sequences in receptors on the cell membrane in the first step of CCVs formation (Van Minnebruggen et al., 2004), so the absence of μ disrupts this process. A previous study also revealed the colocalization of glycoprotein gB (glycoprotein that contains internalization motifs) of PRV with AP-2 complex, demonstrating that during the early steps of the infection, PRV glycoprotein colocalize with clathrin triskelions (Van Minnebruggen et al., 2004).

In addition, no observable differences in shape or size between PK15 and AP2M1-KD were noticed, so cells might use CIE routes to maintain a normal surface/volume ratio. It must be considered that internalization through CME represents a major, albeit not exclusive pathway of entry into cells, as CIE pathways remain active to maintain internalization. That explains that PRV-XGF-N internalization is not completely stopped when blocking CME, taking into account that this pathway is not 100% blocked. In addition, several studies have postulated that clathrin not only plays an important role in the entry of numerous viruses into the host cell, but may also be involved, together with the protein dynamin, in endomembrane vesicular transport and envelope protein delivery to assembly sites, especially in the case of HSV-1 (Albecka et al., 2016). To make sure that in our experiments the decrease of infection when CME is inhibited is only due to blocked entry, infection experiments at short times were performed, evaluating the expression of IE180. Stopping the infection at such short times in experiments from the Figures 2, 4, 5 ensures that the virus has just entered and is performing its first infection cycle; therefore, it has not yet reached the assembly phase where clathrin possibly also plays a transporter role, although potentially to a lesser extent than during entry.

For best visualization of PRV-XGF-N using CME to enter PK15 cells, TEM was chosen as it currently is the unique imaging technique that allows direct visualization of viral particles at a nanometer-scale resolution (Roingeard et al., 2019). The images show virions internalized as cargo in CCVs, and virions surrounded by clathrin, as expected because viruses can produce signals for clathrin recruitment when internalization (Praena et al., 2020). It should be noted that the recombinant PRV-XGF-N strain used in the study lacks the gene encoding the gG glycoprotein. However, this is not of concern for clathrin entry studies, since gG is not essential for virulence and is not part of the virion structure, as it is secreted into the medium by the infected cells (Viejo-Borbolla et al., 2010). Having a GFP-expressing PRV strain that mimics the entry of wild-type PRV certainly speeds up and facilitates experimental work.

Understanding the mechanisms of PRV viral entry into host cells is crucial to contribute to the design of antiviral drugs or strategies that target this early step of infection. Various studies are already proposing antiviral agents against PRV which act early in its viral cycle, such as valpromide (Andreu et al., 2021), valnoctamide (Praena et al., 2019), U18666A (Li et al., 2022), or quercetin (Sun et al., 2021).

Together, our data enrich previous information about PRV viral cycle, focusing on the characterization of viral entry by using CME. We suggest that clathrin plays a relevant role in the endocytic entry of PRV-XGF-N into PK15 swine cell line, as demonstrated in AP2M1-KD cells and in the treatment with CME-inhibitory drugs. Both approaches led to a decrease in PRV-XGF-N infection. Further research not only *in vitro* but in models closer to the clinic is needed to unravel the intracellular trafficking route and the degree of involvement of clathrin and AP-2 in the route of entry of PRV.

## Data availability statement

The original contributions presented in the study are included in the article/Supplementary material, further inquiries can be directed to the corresponding authors.

## Ethics statement

Ethical approval was not required for the studies on animals in accordance with the local legislation and institutional requirements because only commercially available established cell lines were used.

## Author contributions

SA: Data curation, Formal analysis, Investigation, Methodology, Writing – original draft, Writing – review and editing, Conceptualization. CA: Data curation, Investigation, Methodology, Writing – review and editing. IR: Writing – review and editing. JL-G: Conceptualization, Funding acquisition, Project administration, Supervision, Writing – review and editing. RB-M: Conceptualization, Data curation, Formal analysis, Funding acquisition, Investigation, Methodology, Supervision, Writing – original draft, Writing – review and editing.
