# Supplementary material for: Pseudorabies virus uses clathrin mediated endocytosis to enter PK15 swine cell line
Source: Front Microbiol. 2024 Feb 5;15:1332175. doi: 10.3389/fmicb.2024.1332175 (PMC10876092; doi:10.3389/fmicb.2024.1332175)
Supplement: Supplementary file 1 [file Data_Sheet_1.DOCX]

**Supplemental information**

**
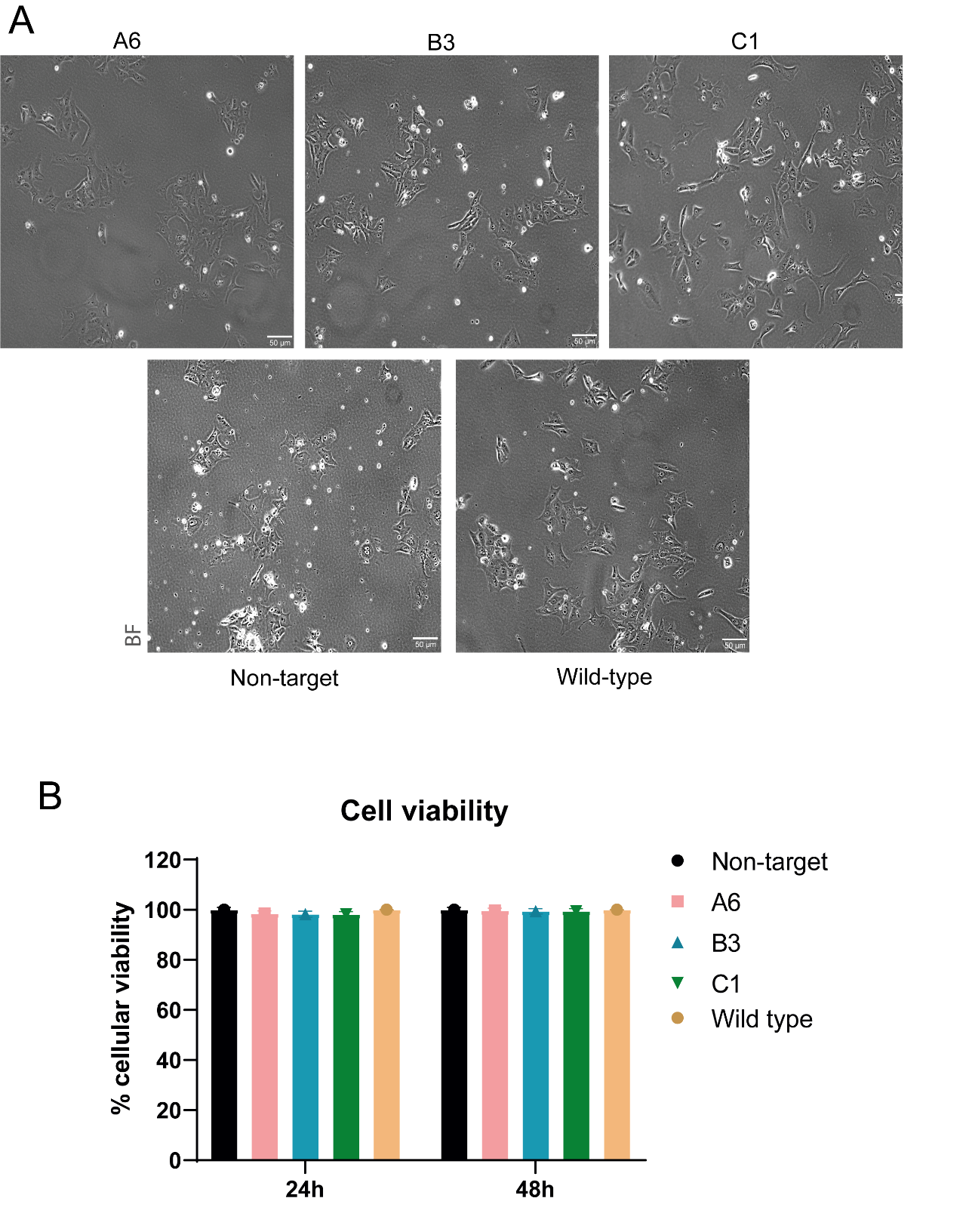
**

**S1- A) Morphology of AP2M1-KD PK15 cells.** Brightfield microscopy images of wild-type PK15 cells, non-target, and AP2M1-KD cells A6, B3, C1. There are no observable differences in size, shape, or volume between them. **B)** **Cell viability of wild-type PK15 cells, non-target, and AP2M1-KD cells A6, B3, C1**. Viability was measured by MTT tetrazolium salt assay and represented as the mean percentage of cellular viability ± S.D. (*n* = 4) after 24 or 48h.

**
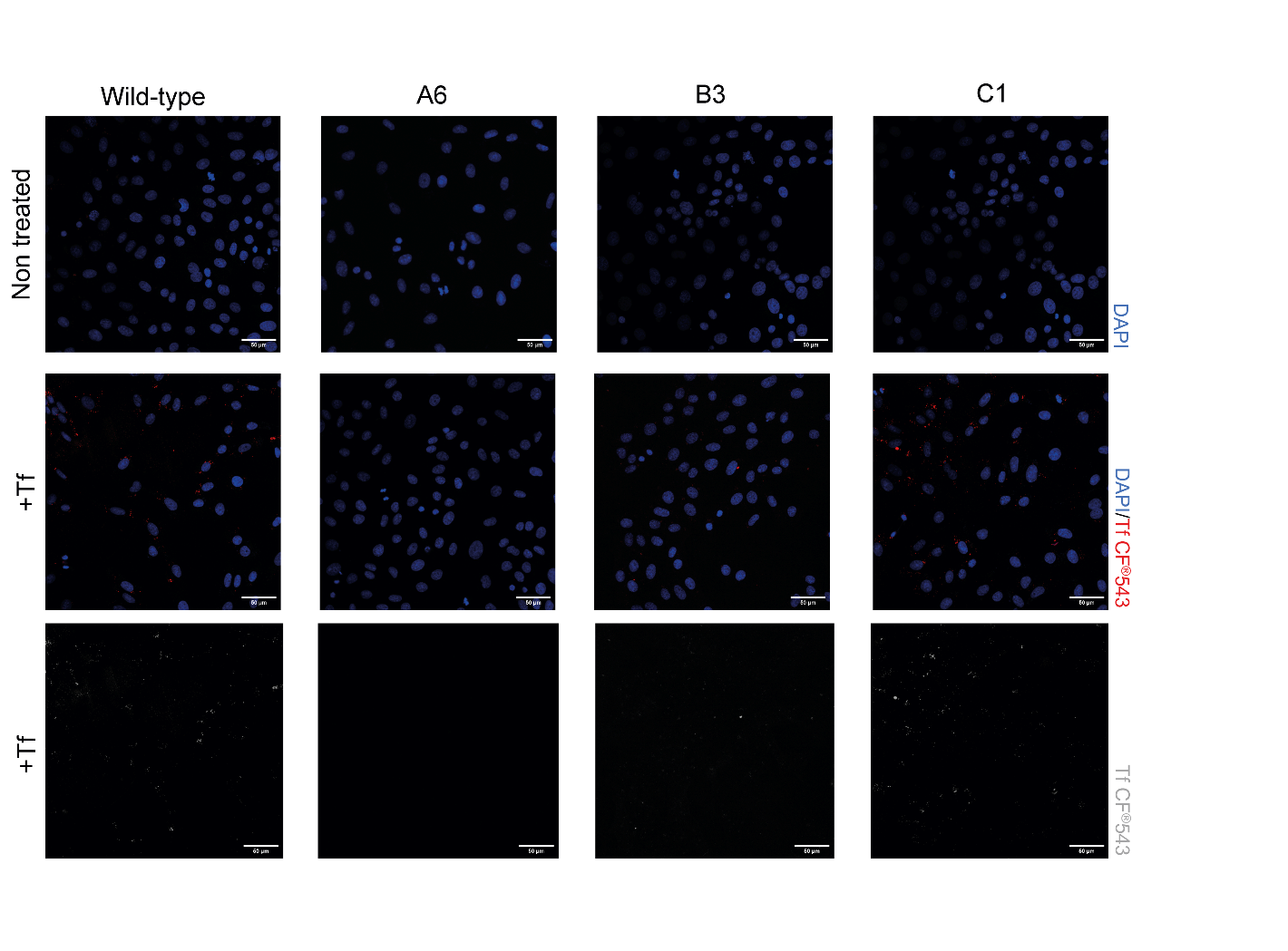
S2- Internalization of transferrin CF®543 is blocked in PK15 successfully AP2M1 knockdown cells.** Wild-type PK15 cells and AP2M1 KD-cells A6, B3 and C1 were treated with labeled Tf CF®543 (5 µg/mL) for 30 min at 4°C. All cells were incubated again for 5 min at 37 °C in a humidified 5% CO_2_ atmosphere to allow internalization of Tf conjugate. Finally, cells were washed with PBS followed by 4% PFA fixation and DAPI staining to reveal nuclei. Scale bar = 50 µm.

**Figure S3. The knockdown of *AP2M1* in PK15 cells reduces wild-type NIA-3 PRV infection.** KD PK15 cells A6, B3, and C1, and non-target cells were subjected to infection with wild-type PK15 NIA-3 strain at an m.o.i of 0.5. At 24 h p.i. cells were collected and titration was made by endpoint dilution assay. Graph shows show the logarithm of viral production titration (TCID_50_/ml) ± S.D. (*n* =3), determined by the Reed and Muench method.
